# Donor-derived cell-free DNA as a diagnostic tool in transplantation

**DOI:** 10.3389/fgene.2022.1031894

**Published:** 2022-10-21

**Authors:** Michael Oellerich, Klemens Budde, Bilgin Osmanodja, Kirsten Bornemann-Kolatzki, Julia Beck, Ekkehard Schütz, Philip D. Walson

**Affiliations:** ^1^ Department of Clinical Pharmacology, University Medical Center Göttingen, Göttingen, Germany; ^2^ Department of Nephrology and Intensive Care, Charité—Universitätsmedizin Berlin, Corporate Member of Freie Universität Berlin, Humboldt-Universität zu Berlin, and Berlin Institute of Health, Berlin, Germany; ^3^ Chronix Biomedical GmbH, Göttingen, Germany

**Keywords:** donor-derived cell-free DNA, transplant surveillance, graft injury, personalized immunosuppression, liquid biopsy

## Abstract

There is a need to improve personalized immunosuppression in organ transplantation to reduce premature graft loss. Biomarkers are needed to better detect rejection, asymptomatic graft injury, and under-immunosuppression. Assessment of minimal necessary exposure to guide tapering and prevent immune activation is also important. There is robust clinical evidence from a large number of published studies supporting the role of dd-cfDNA for monitoring graft integrity and detection or exclusion of rejection. Dd-cfDNA indicates graft cell death without being rejection specific. It can be determined in plasma through droplet digital PCR using preselected SNPs or next generation sequencing. Changes in recipient cfDNA (e.g., by infection) can affect the results of dd-cfDNA fractional determination. This limitation can be overcome using absolute dd-cfDNA quantification. The combination of fractional and absolute determination including total cfDNA is recommended for meaningful interpretation of the results. The value proposition for the patient includes earlier transplant injury detection and intervention, less full blown rejection risk, an alternative to invasive biopsies, and personalized immunosuppression with potential for improved long-term outcome. Transplant physicians benefit from better immunosuppressive guidance and having an alternative when biopsies are refused or contraindicated. Further advantages are improved biopsy interpretation, less trial and error changes in immunosuppression, and less time dealing with complications. The laboratory medicine specialist can provide more effective services. Hospital management and insurance companies could benefit from more cost-effective surveillance of transplant recipients. Potential cost savings would result from fewer biopsies as a result of the tests’ high negative predictive value, fewer re-transplantations, and less organ failure with return to dialysis. A pathway to implementation and metrics is suggested to measure the effectiveness of dd-cfDNA testing.

## Introduction

Improvement of personalized immunosuppression is necessary in kidney transplantation to reduce premature graft loss (Oellerich et al., 2021). Therefore, more efficient biomarkers are needed providing actionable information regarding early detection or exclusion of rejection, asymptomatic graft injury and under-immunosuppression (Brunet et al., 2016). The ability to assess minimal necessary exposure would also be important to guide tapering and to prevent immune activation.

Current approaches used for kidney recipient surveillance, including plasma creatinine, immunosuppressive drug levels, proteinuria, hematuria, donor-specific antibodies (DSA) and duplex ultrasonography, have diagnostic limitations (Brunet et al., 2019; Bergan et al., 2021; Oellerich et al., 2021). Plasma creatinine is a measure of kidney function rather than graft injury. An increase of plasma creatinine is not specific for allograft injury [e.g., can increase due to volume depletion or use of angiotensin converting enzyme (ACE) inhibitors]. Elevated plasma creatinine levels may therefore trigger unnecessary biopsies (Oellerich et al., 2019). Furthermore a significant degree of graft damage may already be present by the time a rise in plasma creatinine is evident (American Society of Nephrology, 2005). Interventions based on plasma creatinine may therefore be too late.

Immunosuppressive drug monitoring indicates the risk of toxicity, but is a poor predictor of graft damage (First et al., 2017). Biopsies can only be performed infrequently and have a major complication rate of 1%. Inadequate specimens are obtained in around 25% of biopsies and interobserver variability of interpretation limits their usefulness (Knight et al., 2019; Oellerich et al., 2019). There is a need for a noninvasive blood-based alternative to surveillance biopsies to rule out “silent” subclinical rejection (Lee et al., 2020). Monitoring the adequacy of immunosuppression is also important as subclinical histologic abnormalities are associated with the development of chronic injury.

### Rationale for using donor-derived cell-free DNA as a biomarker

Against this background noninvasive donor-derived cell-free DNA (dd-cfDNA) determination has great potential to early detect graft injury and/or rejection. The rationale for using dd-cfDNA as a quantitative biomarker (“liquid biopsy”) in transplantation is based on the fact that organ transplants are also genome transplants which opens up the possibility to use them to directly monitor allograft health (De Vlaminck et al., 2014). In the case of graft cell death nucleosomes are released into the bloodstream as cfDNA. Causes of graft injury include rejection, acute tubular necrosis, ischemia, trauma and infection. The mechanisms of cfDNA release involve necrosis yielding larger fragments (∼10,000 bp) or apoptosis resulting in the release of smaller fragments (60–500 bp). The median half-life of released DNA in the circulation is only about 30 min to 2 h (Sherwood and Weimer, 2018). In the recipient´s blood dd-cfDNA accounts for only a small fraction of the total cfDNA. About 90% of cfDNA in the recipient plasma stems from white blood cells undergoing natural apoptosis (Sun et al., 2015). dd-cfDNA fragments also appear in urine (Sigdel et al., 2013) as they are filtered through the glomerular barrier. Moreover dd-cfDNA derived from the donor-derived urinary tract is also excreted in the urine (Oellerich et al., 2021).

### Methods for donor-derived cell-free DNA determination

dd-cfDNA quantification is based on genetic markers (e.g., single-nucleotide polymorphisms or SNPs) that allow discrimination between heterologous alleles in the recipient and donor (Oellerich et al., 2021). Currently in clinical assays either random (Snyder et al., 2011; Sharon et al., 2017) or targeted (Beck et al., 2013; Grskovic et al., 2016; Sigdel et al., 2019) approaches are used. Random approaches use adapter ligation followed by next generation sequencing (NGS). Targeted approaches use preselected SNPs in droplet digital PCR (ddPCR) (Beck et al., 2013) or targeted NGS methods (Grskovic et al., 2016; Sigdel et al., 2019). PCR efficiency is affected by the length of amplicons and cfDNA fragments (Dauber et al., 2020; Oellerich et al., 2021). Recently a dd-cfDNA assay has been evaluated based on donor-recipient HLA-mismatch (human leukocyte antigen) at the *HLA-DRB1* locus using optimized droplet-digital PCR (Sorbini et al., 2021). Fractional determination (%) is possible with all these methods but absolute quantification which is not affected by changes in recipient cfDNA (e.g. by infection) has only been validated for ddPCR (Oellerich et al., 2019; Whitlam et al., 2019). Most of these methods have a lower limit of quantification of ∼0.15% dd-cfDNA and an imprecision (CV) of 3%–12% (Oellerich et al., 2020). Studies in heart transplant recipients reported also lower baseline levels of 0.02% (Agbor-Enoh et al., 2021a), 0.04% (Kim et al., 2022) and 0.07% (Khush et al., 2019). In kidney transplantation a lower limit of 0.08% has recently be published (Halloran et al., 2022). Samples are stable at room temperature for ∼1 week if collected in appropriate tubes (Oellerich et al., 2020).

### Clinical validity of donor-derived cell-free DNA in transplantation

The clinical validity of dd-cfDNA in kidney transplantation has been documented in more than 20 studies (Knight et al., 2019; Wijtvliet et al., 2020; Filippone and Farber, 2021; Oellerich et al., 2021; Xiao et al., 2021; Steggerda et al., 2022). Over the first 2 weeks after transplantation there is typically an exponential decline to a baseline level (Oellerich et al., 2019) which can be used to discriminate graft injury. An abnormal non-exponential decline of dd-cfDNA has been observed in some patients due to urinary tract infections, hemodynamic problems, or surgical complications (Gielis et al., 2018). Most studies investigating dd-cfDNA (%) used a cut-off between 0.74% and 1.0% (Oellerich et al., 2021). Based on simultaneous maximization of sensitivity and specificity threshold values of 0.43% were found for fractional determination and 52 cp/ml for appropriate absolute quantification (Oellerich et al., 2019). Although different concepts have been used to establish threshold values for detection of rejection median dd-cfDNA values in reference populations were very similar ranging from 0.21% to 0.40% (Bloom et al., 2017; Oellerich et al., 2019; Sigdel et al., 2019; Oellerich et al., 2021).

During long-term surveillance a time-dependent increase of dd-cfDNA (%) from 0.8% to 2.1% (90th percentile) was observed as a result of a systematic concurrent decrease of total cfDNA, presumably due to a decrease of calcineurin inhibitor exposure over time (Schuetz et al., 2020). The resulting time-dependent increase of fractional dd-cfDNA (%) brings the use of a single cut-off value during long term treatment into question (Schuetz et al., 2020). In contrast to fractional dd-cfDNA (%) the threshold for absolute quantification (cp/ml) was not affected during long term surveillance (Schuetz et al., 2020).

In patients with either T-cell mediated rejection (TCMR) or antibody-mediated rejection (ABMR) elevations of dd-cfDNA were observed in the vast majority of studies (Knight et al., 2019; Sigdel et al., 2019; Oellerich et al., 2021; Halloran et al., 2022). ABMR is associated with 20%–30% allograft loss (Kim et al., 2014). Early and subclinical ABMR detection enables earlier adapted therapeutic interventions and may improve outcomes (Mayer et al., 2022).

In DSA + patients early detection of ABMR is important and dd-cfDNA may also be helpful to identify patients with “indolent” DSA from those with graft injury due to DSA (Kataria et al., 2021). A clinical trial to prove this hypothesis (NCT04897438) is under way. Measuring dd-cfDNA is also of interest for monitoring the potential therapeutic effect on organ injury of novel ABMR treatments, such as anti-CD38 monoclonal antibody Felzertamab (Mayer et al., 2022).

In the multicenter DART study (Bloom et al., 2017) acute and chronic ABMR could be very well detected by measuring dd-cfDNA (%). For TCMR dd-cfDNA elevations were insufficiently detected with the assay employed. This has been confirmed in an independent study (Huang et al., 2019). The observed false negative TCMR results might have been due to an insufficient detection of short DNA fragments due to the use of relatively long amplicons (100–130 bp) in the particular test used in these two studies (Grskovic et al., 2016). It is possible that in TCMR, which is primarily a process of the kidney tubulointerstitial space, there is more extensive fragmentation of dd-cfDNA compared to ABMR. This hypothesis needs to be confirmed by further investigations.

It has been demonstrated using INDEL q PCR that dd-cfDNA quantification can be substantially improved using smaller amplicon sizes (Dauber et al., 2020). In a further retrospective study using a SNP- based multiplex PCR NGS test TCMR (tubulitis score >2, interstitial inflammation score >2) could be very well detected (Sigdel et al., 2019). Using the same method, in the recent prospective Trifecta multicenter study Halloran et al. (Halloran et al., 2022) compared fractional dd-cfDNA levels to indication biopsy results obtained by a microarray-based Molecular Microscope Diagnostic System (MMDx) and conventional histology. Dd-cfDNA was strongly associated with both molecular and histologic rejection results suggesting the potential of such testing to reduce unnecessary biopsies. Absolute quantification also discriminated very well between patients with TCMR, ABMR, borderline TCMR and acute tubular necrosis from stable and biopsy negative patients (Table 1). Diagnostic accuracy (AUC-ROC) regarding detection of acute rejection was superior for absolute quantification (83%) compared to fractional dd-cfDNA determination (73%) (Oellerich et al., 2019).

**TABLE 1:** Detection of rejection by absolute dd-cfDNA quantification in the first year post-KTx.

Prospective single center trial using ddPCR			
Histopathology findings	N	n	dd-cfDNA (cp/ml) Median (IQR)
Biopsy proven acute rejection (TCMR, ABMR)	15	22	82 (53–147)*
ATN	29	32	64 (43–126)**
IFTA	24	30	35 (23–84)***
Stable Phase (rejection-free) (SP)	83	408	25 (11–60)

* *p* < 0.0001 vs. SP **p = 0.0001 vs. SP ***p = 0.02 vs. SP cut-off: 52 cp/ml. Data from Ref. (Oellerich et al., 2019).

A problem with fractional dd-cfDNA determination is that it is influenced by variations in total cf-DNA levels due to leukopenia, leukocytosis and inflammatory illness; leading to falsely elevated or decreased results (Whitlam et al., 2019; Osmanodja et al., 2021). Of particular importance is the systematic increase of dd-cfDNA percentages when CNI are tapered, due to an influence of CNI on leucocyte stability (Schuetz et al., 2020). In clinical practice a combination of absolute quantification and fractional abundance of dd-cfDNA with consideration of total cfDNA as well seems to provide the most comprehensive diagnostic information (Oellerich et al., 2019; Whitlam et al., 2019; Oellerich et al., 2021; Osmanodja et al., 2021).

The overall diagnostic performance of dd-cfDNA for detection of rejection after KTx seems to be adequate based on data reported to date (Oellerich et al., 2021). Based on pooled data from published studies the diagnostic accuracy (ROC AUC) was on average 0.81 (seven studies) for dd-cfDNA (%) and 0.87 (two studies) for dd-cfDNA (cp/ml) (Oellerich et al., 2021). Mean values for sensitivity were 80% for dd-cfDNA (%) and 79% for dd-cfDNA (cp/ml). For specificity, mean values were 76% for both dd-cfDNA (%) and (cp/ml). Positive predictive values (that depend on the variable prevalence of rejection in the study populations) were 56% for dd-cfDNA (%) and 33% for dd-cfDNA (cp/ml). The very high negative predictive values of 90% for dd-cfDNA (%) and 97% for dd-cfDNA (cp/ml) indicate that this test is useful to exclude rejections with high probability. Still, other entities that can cause elevations such as recurrent primary disease may be excluded with high probability only by kidney allograft biopsy. (Oellerich et al., 2021).

Dd-cfDNA detects rejection also in liver (Schuetz et al., 2017; Levitsky et al., 2022), heart (Snyder et al., 2011; De Vlaminck et al., 2014; Khush et al., 2019; Richmond et al., 2020; Agbor-Enoh et al., 2021a; Sorbini et al., 2021), lung (Rosenheck et al., 2022a) and simultaneous pancreas-kidney (Ventura-Aguiar et al., 2022) transplant patients with good accuracy (Table 2). Dd-cfDNA fraction was also significantly elevated for CLAD (De Vlaminck et al., 2015). The high negative predictive value of 97% in heart transplant recipients (Khush et al., 2019; Kim et al., 2022; Knuettgen et al., 2022) has the potential to reduce the number of endomyocardial biopsies that have a serious complication rate of 0.5%–1% (Miller et al., 2013; Knuettgen et al., 2022).

**TABLE 2:** Diagnostic performance of plasma dd-cfDNA for detection of rejection in solid organ transplantation.

Study	Organ	Threshold dd-cfDNA	ROC-AUC	Sensitivity %	Specificity %	PPV^a^ %	NPV^a^ %
Bloom 2017 Bloom et al. (2017)	Kidney	1.00%	0.74	59	85	61	84
Sigdel 2019 Sigdel et al. (2019)	Kidney	1.00%	0.87	89	73	52	95
Whitlam 2019 Whitlam et al. (2019)	Kidney	0.75%	0.89	85	75	48	95
		13 cp/ml	0.91	85	79	52	95
Oellerich 2019 Oellerich et al. (2019)	Kidney	0.43%	0.73	73	69	12	98
		52 cp/ml	0.83	73	73	13	98
Huang 2019 Huang et al. (2019)	Kidney	0.74%	0.71	79	72	77	75
Dauber 2020 Dauber et al. (2020)	Kidney	2.7%	0.84	88	81	64	94
Zhang 2020 Zhang et al. (2020)	Kidney	1.0%	0.90	89	74	76	88
De Vlaminick 2014 De Vlaminck et al. (2014)	Heart	0.25%	0.83	58	93	NR	NR
Richmond 2020 (Richmond et al. (2020)	Heart	0.30%	0.78	60	92	80	81
Khush 2019 Khush et al. (2019)	Heart	0.20%	0.64	44	80	9	97
Agbor-Enoh 2021 Agbor-Enoh et al. (2021a)	Heart	0.25%	0.92	81	85	20	99
Knüttgen 2022 Knuettgen et al. (2022)	Heart	0.35%	0.81	76	83	31	97
Kim 2022 Kim et al. (2022)	Heart	0.15%	0.86	79	77	25	97
Sorbini 2021 Sorbini et al. (2021)	Heart	0.11%	0.72	64	71	NR	NR
De Vlaminck 2015 De Vlaminck et al. (2015)	Lung	1.0%	0.90	100	73	NR	NR
Sayah 2020 Sayah et al. (2020)	Lung	0.87%	0.72	73	53	34	86
Khush 2021 Khush et al. (2021)	Lung	0.85%	0.67	56	76	43	84
Jang 2021 Jang et al. (2021)	Lung	1.0%	0.89	77	84	60	90
Keller 2022 Keller and Agbor-Enoh, (2022)	Lung	1.0%	0.79	76	70	NR	NR
Rosenheck 2022 Rosenheck et al. (2022b)	Lung	1.0%	0.91	89	83	52	97
Schütz 2017 Schuetz et al. (2017)	Liver	10%	0.97	90	93	NR	NR
Levitsky 2021 Levitsky et al. (2022)	Liver	5%	0.95	100	80	63	100
Baumann 2022 Baumann et al. (2022)	Liver	10%	0.92	81	91	NR	NR
Ventura-Aguiar 2022 Ventura-Aguiar et al. (2022)	Pancreas-kidney	10%	0.84	29	100	NR	NR
		70 cp/ml	0.89	86	94	86	94

NR, not reported.

^a^ Predictive values also depend on the variable prevalence of rejection in the study populations. Examples for variable acute rejection incidence are given in Ref (Kataria et al., 2021).

Monitoring of dd-cfDNA may also be helpful to guide immunosuppression minimization. Increased dd-cfDNA values were associated with subtherapeutic tacrolimus concentrations after kidney transplantation in patients with variable tacrolimus levels (Oellerich et al., 2019). Therefore dd-cfDNA might be helpful to detect subclinical graft damage caused by immune activation due to under-immunosuppression (Oellerich et al., 2021). This would be very useful in kidney recipients with a high epitope mismatch burden, high immune competence or non-adherence.

In a recent study, fractional dd-cfDNA was tested during subclinical graft injury after liver transplantation confirmed by surveillance biopsies (Baumann et al., 2022). The lower limit of quantification was 0.15% and the interassay CV ranged from 3% to 5.4%. Dd-cfDNA increased stepwise significantly from patients without histological signs of rejection (median, 2.2%) over subclinical TCMR (median, 3.5%) to clinical overt TCMR (median, 23%). The data confirmed the 10% cut-off for liver (Schuetz et al., 2017) yielding a similar sensitivity and specificity for acute rejection. Before immunosuppression minimization subclinical TCMR should be excluded. The usefulness of longitudinal dd-cfDNA monitoring of subclinical injury should be assessed in future prospective studies.

A majority of studies have found that dd-cfDNA was clearly superior compared with eGFR in the discrimination of patients with or without acute rejection (Bloom et al., 2017; Sigdel et al., 2019; Whitlam et al., 2019; Oellerich et al., 2021). Dd-cfDNA release, however, is not rejection-specific and elevations can also be observed due to other causes of graft injury (Bloom et al., 2017; Oellerich et al., 2021). Elevated dd-cfDNA levels have also been found in patients with pyelonephritis and biopsy-proven BKV nephropathy (Sigdel et al., 2013; Bloom et al., 2017; Oellerich et al., 2021). In particular, urinary dd-cfDNA concentration was significantly elevated in patients with BKV nephropathy (Sigdel et al., 2013; Chen et al., 2022). In patients with interstitial fibrosis/tubular atrophy (IFTA) only a small increase of dd-cfDNA was observed (Sigdel et al., 2013; Bloom et al., 2017; Oellerich et al., 2019; Whitlam et al., 2019) potentially due to lower viable cell numbers. Due to the slow fibrosing process IFTA may not be associated with a relevant release of DNA from slowly dying kidney cells and the resulting gradual decrease in viable cells over time. During stages of active disease progress, however, dd-cfDNA values might be increased in IFTA.

### Benefits of donor-derived cell-free DNA testing

A previously published value proposition concept (Price et al., 2016) can be used to characterize the benefits of dd-cfDNA-testing to stakeholders involved in organ transplantation (Oellerich et al., 2020).

Various stakeholders are involved in delivering and receiving care in transplantation. There are the transplant patients whose care could be altered by the less invasive graft injury detection, clinicians who manage solid organ transplant patients, laboratory medicine specialists who analyze and interpret test results and hospital management, insurance companies, public payers and policy makers who are involved in providing value-based health care. Value in this context is defined as outcomes relative to costs. For patients the following benefits are expected relative to outcome: detection or exclusion of graft injury or rejection, earlier transplant injury detection and subsequent intervention, biopsy alternative, early diagnosis of subclinical antibody-mediated rejection, detection of under-immunosuppression, evaluation of infectious complications of kidney allografts and personalized immunosuppression. Benefits for transplant physicians include better personalized immunosuppression guidance; for example during tapering, enhanced biopsy interpretation, less trial and error changing of immunosuppression, less time dealing with complications, and indication of response to rejection treatment. Laboratory medicine specialists would have increased involvement in molecular diagnostics regarding the use and interpretation of tests. Hospital management, insurance companies, public payers and policy makers can expect cost savings due to a decreased burden for care-givers; for example due to fewer re-transplantations or less returns to dialysis in KTx.

Dd-cfDNA has great potential for cost savings. Acute rejection is associated with a significant increase in the cost of care (17,000–28,000 USD/per year) (First et al., 2017). TCMR and ABMR contributed to graft loss in more than 60% of graft failures (Mayrdorfer et al., 2021). A graft failure with return to dialysis causes an average annual expense of about 75,000 USD. If the patient is retransplanted, the average cost is about 111,000 USD. The annualized cost of transplantation is less than 25% of the cost of dialysis (16,844 USD vs. 70,581 USD) (First et al., 2017). Compared to these costs dd-cfDNA testing by ddPCR is reasonable with about 401 USD per test based on the German Code of Medical Fees (GOÄ) (Oellerich et al., 2021). As these data show, improvement of outcome in transplantation could save substantial costs.

For implementation of dd-cfDNA in clinical practice the following points are relevant. Depending on the required turnaround time, transplant centers can either set up the test themselves or utilize a qualified external central laboratory. Using ddPCR results can be obtained in about 1 working day and by the use of NGS in 2-3 working days (Oellerich et al., 2021). Implementation within a laboratory requires the availability of appropriate instrumentation (e.g., Illumina NGS instrument or Biorad ddPCR). Qualified laboratory medicine specialists and a quality management program are also necessary. Assessment of clinical utility in prospective randomized outcome studies and the development of clinical practice recommendations are desirable. Metrics for monitoring the impact of implementing the test are summarized in Table 3.

**TABLE 3:** Metrics for monitoring impact of implementation (Oellerich et al., 2020).


- Decrease in number of unnecessary biopsies
- Increase in rate of pathology findings requiring treatment
- Influence on number of acute or chronic rejections
- Number of patients successfully tapered
- Decrease in number of patients with underimmunosuppression and dnDSA formation
- Improvement of clinical outcomes (e.g. decrease of premature graft loss)
- Reduction of overall costs of care

## Conclusion and future perspectives

Overall, evidence regarding clinical validity suggests that dd-cfDNA monitoring can facilitate personalized immunosuppression and thereby potentially decrease premature graft loss and health care costs (Knight et al., 2019; Agbor-Enoh et al., 2021b; Filippone and Farber, 2021; Oellerich et al., 2021). Therefore, Medicare already provides coverage for dd-cfDNA routine testing in the US. The test provides actionable information for early detection or exclusion of rejection. It may facilitate the detection of under-immunosuppression and guide immunosuppression minimization. Clinical utility is currently being studied (NCT 03326076) in kidney transplant recipients, where clinical outcomes in patients monitored with dd-cfDNA will be compared with a matched control cohort without monitoring (Knight et al., 2019). In another study (Precision Medicine can PREVENT AMR: Applying precision medicine technologies in Canada to prevent antibody mediated rejection and premature kidney transplant loss) dd-cfDNA is being tested in combination with other immunological strategies. These include HLA epitope donor matching, T cell receptor sequencing, deep cytometric phenotyping of immune cell subsets and routine DSA monitoring, to achieve personalized immunosuppression. A combination of dd-cfDNA with conventional and other novel biomarkers, such as chemokines CXCL9 und CXCL10 (Nolan et al., 2020; Yang et al., 2020) should be further explored. Studies on micro RNAs are in an exploratory phase and combinations may improve clinical usefulness for detection of rejection in kidney transplantation (Matz et al., 2016; Ledeganck et al., 2019). In addition, immune repertoire sequencing has potential for post-transplant immune monitoring. Future studies may test whether a combination of dd-cfDNA serial determinations with immune monitoring (T cell receptor sequencing, activated B cell sequencing) could facilitate early detection of immune activation resulting in graft damage (Alachkar et al., 2016; Pineda et al., 2019). It is expected that all of these studies will provide insights into the impact of dd-cfDNA monitoring on clinical outcomes.

Integration of all available data on relevant clinical and diagnostic findings with dd-cfDNA results should enable individualized transplant patient therapy (Oellerich et al., 2021).

## Abbreviations

ABMR, Antibody-mediated rejection; AR, Acute rejection; BKV, BK polyomavirus; BKV N, BK polyomavirus-associated nephropathy; cfDNA, cell-free DNA; CLAD, Chronic lung allograft dysfunction; CXCL10, CXC-chemokine ligand 10; CXCL9, CXC-chemokine ligand 9; dd-cfDNA, Donor-derived cell-free DNA; ddPCR, Droplet digital PCR; DSA, Donor-specific antibodies; HLA, Human leukocyte antigen; IFTA, Interstitial fibrosis/tubular atrophy; INDEL, Insertion/deletion; MMDx, Molecular Microscope Diagnostic System; NGS, Next-generation sequencing; NPV, Negative predictive value; PCR, Polymerase chain reaction; PPV, Positive predictive value; ROC AUC, Receiver operating characteristic area under the curve; SNP, Single nucleotide polymorphism; TCMR, T-cell mediated rejection.

## References

[B1] Agbor-Enoh S. Oellerich M. Wu A. Halloran P. F. De Vlaminck I. Keller M. (2021). Molecular approaches to transplant monitoring; is the horizon here? Clin. Chem. 67, 1443–1449. 10.1093/clinchem/hvab183 34632495

[B2] Agbor-Enoh S. Shah P. Tunc I. Hsu S. Russell S. Feller E. (2021). Cell-free DNA to detect heart allograft acute rejection. Circulation 143, 1184–1197. 10.1161/CIRCULATIONAHA.120.049098 33435695 PMC8221834

[B3] Alachkar H. Mutonga M. Kato T. Kalluri S. Kakuta Y. Uemura M. (2016). Quantitative characterization of T-cell repertoire and biomarkers in kidney transplant rejection. BMC Nephrol. 17, 181. 10.1186/s12882-016-0395-3 27871261 PMC5117555

[B4] American Society of Nephrology (2005). American society of Nephrology renal research report. J. Am. Soc. Nephrol. 16, 1886–1903. 10.1681/ASN.2005030285 15888557

[B5] Baumann A. K. Beck J. Kirchner T. Hartleben B. Schutz E. Oellerich M. (2022). Elevated fractional donor-derived cell-free DNA during subclinical graft injury after liver transplantation. Liver Transplant. 10.1002/lt.26479 35429207

[B6] Beck J. Bierau S. Balzer S. Andag R. Kanzow P. Schmitz J. (2013). Digital droplet PCR for rapid quantification of donor DNA in the circulation of transplant recipients as a potential universal biomarker of graft injury. Clin. Chem. 59, 1732–1741. 10.1373/clinchem.2013.210328 24061615

[B7] Bergan S. Brunet M. Hesselink D. A. Johnson-Davis K. L. Kunicki P. K. Lemaitre F. (2021). Personalized therapy for mycophenolate: Consensus report by the international association of therapeutic drug monitoring and clinical toxicology. Ther. Drug Monit. 43, 150–200. 10.1097/FTD.0000000000000871 33711005

[B8] Bloom R. D. Bromberg J. S. Poggio E. D. Bunnapradist S. Langone A. J. Sood P. (2017). Cell-free DNA and active rejection in kidney allografts. J. Am. Soc. Nephrol. 28, 2221–2232. 10.1681/ASN.2016091034 28280140 PMC5491290

[B9] Brunet M. Shipkova M. van Gelder T. Wieland E. Sommerer C. Budde K. (2016). Barcelona consensus on biomarker-based immunosuppressive drugs management in solid organ transplantation. Ther. Drug Monit. 38, S1–S20. 10.1097/FTD.0000000000000287 26977997

[B10] Brunet M. van Gelder T. Asberg A. Haufroid V. Hesselink D. A. Langman L. (2019). Therapeutic drug monitoring of tacrolimus-personalized therapy: Second consensus report. Ther. Drug Monit. 41, 261–307. 10.1097/FTD.0000000000000640 31045868

[B11] Chen X. T. Qiu J. Wu Z. X. Zhang H. Chen T. Yang S. C. (2022). Using both plasma and urine donor-derived cell-free DNA to identify various renal allograft injuries. Clin. Chem. 68, 814–825. 10.1093/clinchem/hvac053 35587713

[B12] Dauber E. M. Kollmann D. Kozakowski N. Rasoul-Rockenschaub S. Soliman T. Berlakovich G. A. (2020). Quantitative PCR of INDELs to measure donor-derived cell-free DNA-a potential method to detect acute rejection in kidney transplantation: A pilot study. Transpl. Int. 33, 298–309. 10.1111/tri.13554 31710731 PMC7065216

[B13] De Vlaminck I. Martin L. Kertesz M. Patel K. Kowarsky M. Strehl C. (2015). Noninvasive monitoring of infection and rejection after lung transplantation. Proc. Natl. Acad. Sci. U. S. A. 112, 13336–13341. 10.1073/pnas.1517494112 26460048 PMC4629384

[B14] De Vlaminck I. Valantine H. A. Snyder T. M. Strehl C. Cohen G. Luikart H. (2014). Circulating cell-free DNA enables noninvasive diagnosis of heart transplant rejection. Sci. Transl. Med. 6, 241ra77. 10.1126/scitranslmed.3007803 PMC4326260 24944192

[B15] Filippone E. J. Farber J. L. (2021). The monitoring of donor-derived cell-free DNA in kidney transplantation. Transplantation 105, 509–516. 10.1097/TP.0000000000003393 32732615

[B16] First R. M. Lee D. Lewis P. Rose S. (2017). An economic analysis of the cost effectiveness of blood gene expression profiling in kidney transplant recipients. J. Health Med. Econ. 3, 3. 10.21767/2471-9927.100029

[B17] Gielis E. M. Beirnaert C. Dendooven A. Meysman P. Laukens K. De Schrijver J. (2018). Plasma donor-derived cell-free DNA kinetics after kidney transplantation using a single tube multiplex PCR assay. PLoS One 13, e0208207. 10.1371/journal.pone.0208207 30521549 PMC6283554

[B18] Grskovic M. Hiller D. J. Eubank L. A. Sninsky J. J. Christopherson C. Collins J. P. (2016). Validation of a clinical-grade Assay to measure donor-derived cell-free DNA in solid organ transplant recipients. J. Mol. Diagn. 18, 890–902. 10.1016/j.jmoldx.2016.07.003 27727019

[B19] Halloran P. F. Reeve J. Madill-Thomsen K. S. Demko Z. Prewett A. Billings P. (2022). The Trifecta study: Comparing plasma levels of donor-derived cell-free DNA with the molecular phenotype of kidney transplant biopsies. J. Am. Soc. Nephrol. 33, 387–400. 10.1681/ASN.2021091191 35058354 PMC8819982

[B20] Huang E. Sethi S. Peng A. Najjar R. Mirocha J. Haas M. (2019). Early clinical experience using donor-derived cell-free DNA to detect rejection in kidney transplant recipients. Am. J. Transpl. 19, 1663–1670. 10.1111/ajt.15289 30725531

[B21] Jang M. K. Tunc I. Berry G. J. Marboe C. Kong H. Keller M. B. (2021). Donor-derived cell-free DNA accurately detects acute rejection in lung transplant patients, a multicenter cohort study. J. Heart Lung Transpl. 40, 822–830. 10.1016/j.healun.2021.04.009 PMC8319066 34130911

[B22] Kataria A. Kumar D. Gupta G. (2021). Donor-derived cell-free DNA in solid-organ transplant diagnostics: Indications, limitations, and future directions. Transplantation 105, 1203–1211. 10.1097/TP.0000000000003651 33534526

[B23] Keller M. Agbor-Enoh S. (2022). Cell-free DNA in lung transplantation: Research tool or clinical workhorse? Curr. Opin. Organ Transpl. 27, 177–183. 10.1097/MOT.0000000000000979 PMC9179944 35649108

[B24] Khush K. K. De Vlaminck I. Luikart H. Ross D. J. Nicolls M. R. (2021). Donor-derived, cell-free DNA levels by next-generation targeted sequencing are elevated in allograft rejection after lung transplantation. ERJ Open Res. 7, 00462–02020. 10.1183/23120541.00462-2020 33532456 PMC7836440

[B25] Khush K. K. Patel J. Pinney S. Kao A. Alharethi R. DePasquale E. (2019). Noninvasive detection of graft injury after heart transplant using donor-derived cell-free DNA: A prospective multicenter study. Am. J. Transpl. 19, 2889–2899. 10.1111/ajt.15339 PMC6790566 30835940

[B26] Kim M. Martin S. T. Townsend K. R. Gabardi S. (2014). Antibody-mediated rejection in kidney transplantation: A review of pathophysiology, diagnosis, and treatment options. Pharmacotherapy 34, 733–744. 10.1002/phar.1426 24753207

[B27] Kim P. J. Olymbios M. Siu A. Wever Pinzon O. Adler E. Liang N. (2022). A novel donor-derived cell-free DNA assay for the detection of acute rejection in heart transplantation. J. Heart Lung Transpl. 41, 919–927. 10.1016/j.healun.2022.04.002 PMC9670834 35577713

[B28] Knight S. R. Thorne A. Lo Faro M. L. (2019). Donor-specific cell-free DNA as a biomarker in solid organ transplantation. A systematic review. Transplantation 103, 273–283. 10.1097/TP.0000000000002482 30308576

[B29] Knuettgen F. Beck J. Dittrich M. Oellerich M. Zittermann A. Schulz U. (2022). Graft-derived cell-free DNA as a noninvasive biomarker of cardiac allograft rejection: A cohort study on clinical validity and confounding factors. Transplantation 106, 615–622. 10.1097/TP.0000000000003725 33653997

[B30] Ledeganck K. J. Gielis E. M. Abramowicz D. Stenvinkel P. Shiels P. G. Van Craenenbroeck A. H. (2019). MicroRNAs in AKI and kidney transplantation. Clin. J. Am. Soc. Nephrol. 14, 454–468. 10.2215/CJN.08020718 30602462 PMC6419285

[B31] Lee D. M. Abecassis M. M. Friedewald J. J. Rose S. First M. R. (2020). Kidney graft surveillance biopsy utilization and trends: Results from a survey of high-volume transplant centers. Transpl. Proc. 52, 3085–3089. 10.1016/j.transproceed.2020.04.1816 32576474

[B32] Levitsky J. Kandpal M. Guo K. Kleiboeker S. Sinha R. Abecassis M. (2022). Donor-derived cell-free DNA levels predict graft injury in liver transplant recipients. Am. J. Transpl. 22, 532–540. 10.1111/ajt.16835 34510731

[B33] Matz M. Fabritius K. Lorkowski C. Durr M. Gaedeke J. Durek P. (2016). Identification of T Cell-Mediated vascular rejection after kidney transplantation by the combined measurement of 5 specific MicroRNAs in blood. Transplantation 100, 898–907. 10.1097/TP.0000000000000873 26444957

[B34] Mayer K. A. Budde K. Jilma B. Doberer K. Bohmig G. A. (2022). Emerging drugs for antibody-mediated rejection after kidney transplantation: A focus on phase II & III trials. Expert Opin. Emerg. Drugs 27, 151–167. 10.1080/14728214.2022.2091131 35715978

[B35] Mayrdorfer M. Liefeldt L. Wu K. Rudolph B. Zhang Q. Friedersdorff F. (2021). Exploring the complexity of death-censored kidney allograft failure. J. Am. Soc. Nephrol. 32, 1513–1526. 10.1681/ASN.2020081215 33883251 PMC8259637

[B36] Miller C. A. Fildes J. E. Ray S. G. Doran H. Yonan N. Williams S. G. (2013). Non-invasive approaches for the diagnosis of acute cardiac allograft rejection. Heart 99, 445–453. 10.1136/heartjnl-2012-302759 23257172

[B37] Nolan N. Valdivieso K. Mani R. Yang J. Y. C. Sarwal R. D. Katzenbach P. (2020). Clinical and analytical validation of a novel urine-based test for the detection of allograft rejection in renal transplant patients. J. Clin. Med. 9, E2325. 10.3390/jcm9082325 32707779 PMC7465561

[B38] Oellerich M. Christenson R. H. Beck J. Schutz E. Sherwood K. Price C. P. (2020). Donor-derived cell-free DNA testing in solid organ transplantation: A value proposition. J. Appl. Lab. Med. 5, 993–1004. 10.1093/jalm/jfaa062 32447378

[B39] Oellerich M. Sherwood K. Keown P. Schutz E. Beck J. Stegbauer J. (2021). Liquid biopsies: Donor-derived cell-free DNA for the detection of kidney allograft injury. Nat. Rev. Nephrol. 17, 591–603. 10.1038/s41581-021-00428-0 34031575

[B40] Oellerich M. Shipkova M. Asendorf T. Walson P. D. Schauerte V. Mettenmeyer N. (2019). Absolute quantification of donor-derived cell-free DNA as a marker of rejection and graft injury in kidney transplantation: Results from a prospective observational study. Am. J. Transpl. 19, 3087–3099. 10.1111/ajt.15416 PMC6899936 31062511

[B41] Osmanodja B. Akifova A. Budde K. Choi M. Oellerich M. Schutz E. (2021). Absolute or relative quantification of donor-derived cell-free DNA in kidney transplant recipients: Case series. Transpl. Direct 7, e778. 10.1097/TXD.0000000000001237 PMC8547915 34712778

[B42] Pineda S. Sigdel T. K. Liberto J. M. Vincenti F. Sirota M. Sarwal M. M. (2019). Characterizing pre-transplant and post-transplant kidney rejection risk by B cell immune repertoire sequencing. Nat. Commun. 10, 1906. 10.1038/s41467-019-09930-3 31015506 PMC6479061

[B43] Price C. P. John A. S. Christenson R. Scharnhorst V. Oellerich M. Jones P. (2016). Leveraging the real value of laboratory medicine with the value proposition. Clin. Chim. Acta. 462, 183–186. 10.1016/j.cca.2016.09.006 27649855

[B44] Richmond M. E. Zangwill S. D. Kindel S. J. Deshpande S. R. Schroder J. N. Bichell D. P. (2020). Donor fraction cell-free DNA and rejection in adult and pediatric heart transplantation. J. Heart Lung Transpl. 39, 454–463. 10.1016/j.healun.2019.11.015 31983667

[B45] Rosenheck J. P. Keller B. C. Fehringer G. Demko Z. P. Bohrade S. M. Ross D. J. (2022). Why cell-free DNA can Be a "game changer" for lung allograft monitoring for rejection and infection. Curr. Pulmonol. Rep. 11, 75–85. 10.1007/s13665-022-00292-8 35910533 PMC9315332

[B46] Rosenheck J. P. Ross D. J. Botros M. Wong A. Sternberg J. Chen Y. A. (2022). Clinical validation of a plasma donor-derived cell-free DNA assay to detect allograft rejection and injury in lung transplant. Transpl. Direct 8, e1317. 10.1097/TXD.0000000000001317 PMC8963832 35372675

[B47] Sayah D. Weigt S. S. Ramsey A. Ardehali A. Golden J. Ross D. J. (2020). Plasma donor-derived cell-free DNA levels are increased during acute cellular rejection after lung transplant: Pilot data. Transpl. Direct 6, e608. 10.1097/TXD.0000000000001063 PMC7515612 33062841

[B48] Schuetz E. Asendorf T. Beck J. Schauerte V. Mettenmeyer N. Shipkova M. (2020). Time-dependent apparent increase in dd-cfDNA percentage in clinically stable patients between one and five years following kidney transplantation. Clin. Chem. 66, 1290–1299. 10.1093/clinchem/hvaa175 33001185

[B49] Schuetz E. Fischer A. Beck J. Harden M. Koch M. Wuensch T. (2017). Graft-derived cell-free DNA, a noninvasive early rejection and graft damage marker in liver transplantation: A prospective, observational, multicenter cohort study. PLoS Med. 14, e1002286. 10.1371/journal.pmed.1002286 28441386 PMC5404754

[B50] Sharon E. Shi H. Kharbanda S. Koh W. Martin L. R. Khush K. K. (2017). Quantification of transplant-derived circulating cell-free DNA in absence of a donor genotype. PLoS Comput. Biol. 13, e1005629. 10.1371/journal.pcbi.1005629 28771616 PMC5542400

[B51] Sherwood K. Weimer E. T. (2018). Characteristics, properties, and potential applications of circulating cell-free DNA in clinical diagnostics: A focus on transplantation. J. Immunol. Methods 463, 27–38. 10.1016/j.jim.2018.09.011 30267663

[B52] Sigdel T. K. Archila F. A. Constantin T. Prins S. A. Liberto J. Damm I. (2019). Optimizing detection of kidney transplant injury by assessment of donor-derived cell-free DNA via massively multiplex PCR. J. Clin. Med. 8, 19. 10.3390/jcm8010019 PMC6352163 30583588

[B53] Sigdel T. K. Vitalone M. J. Tran T. Q. Dai H. Hsieh S. C. Salvatierra O. (2013). A rapid noninvasive assay for the detection of renal transplant injury. Transplantation 96, 97–101. 10.1097/TP.0b013e318295ee5a 23756769 PMC4472435

[B54] Snyder T. M. Khush K. K. Valantine H. A. Quake S. R. (2011). Universal noninvasive detection of solid organ transplant rejection. Proc. Natl. Acad. Sci. U. S. A. 108, 6229–6234. 10.1073/pnas.1013924108 21444804 PMC3076856

[B55] Sorbini M. Togliatto G. M. Simonato E. Boffini M. Cappuccio M. Gambella A. (2021). HLA-DRB1 mismatch-based identification of donor-derived cell free DNA (dd-cfDNA) as a marker of rejection in heart transplant recipients: A single-institution pilot study. J. Heart Lung Transpl. 40, 794–804. 10.1016/j.healun.2021.05.001 34134912

[B56] Steggerda J. A. Pizzo H. Garrison J. Zhang X. Haas M. Kim I. K. (2022). Use of a donor-derived cell-free DNA assay to monitor treatment response in pediatric renal transplant recipients with allograft rejection. Pediatr. Transpl. 26, e14258. 10.1111/petr.14258 35340104

[B57] Sun K. Jiang P. Chan K. C. Wong J. Cheng Y. K. Y. Liang R. H. S. (2015). Plasma DNA tissue mapping by genome-wide methylation sequencing for noninvasive prenatal, cancer, and transplantation assessments. Proc. Natl. Acad. Sci. U. S. A. 112, E5503–E5512. 10.1073/pnas.1508736112 26392541 PMC4603482

[B58] Ventura-Aguiar P. Ramirez-Bajo M. J. Rovira J. Banon-Maneus E. Hierro N. Lazo M. (2022). Donor-derived cell-free DNA shows high sensitivity for the diagnosis of pancreas graft rejection in simultaneous pancreas-kidney transplantation. Transplantation 106, 1690–1697. 10.1097/TP.0000000000004088 35289777 PMC9311279

[B59] Whitlam J. B. Ling L. Skene A. Kanellis J. Ierino F. L. Slater H. R. (2019). Diagnostic application of kidney allograft-derived absolute cell-free DNA levels during transplant dysfunction. Am. J. Transpl. 19, 1037–1049. 10.1111/ajt.15142 30312536

[B60] Wijtvliet V. Plaeke P. Abrams S. Hens N. Gielis E. M. Hellemans R. (2020). Donor-derived cell-free DNA as a biomarker for rejection after kidney transplantation: A systematic review and meta-analysis. Transpl. Int. 33, 1626–1642. 10.1111/tri.13753 32981117

[B61] Xiao H. Gao F. Pang Q. Xia Q. Zeng X. Peng J. (2021). Diagnostic accuracy of donor-derived cell-free DNA in renal-allograft rejection: A meta-analysis. Transplantation 105, 1303–1310. 10.1097/TP.0000000000003443 32890130

[B62] Yang J. Y. C. Sarwal R. D. Sigdel T. K. Damm I. Rosenbaum B. Liberto J. M. (2020). A urine score for noninvasive accurate diagnosis and prediction of kidney transplant rejection. Sci. Transl. Med. 12, eaba2501. 10.1126/scitranslmed.aba2501 32188722 PMC8289390

[B63] Zhang H. Zheng C. Li X. Fu Q. Li J. Su Q. (2020). Diagnostic performance of donor-derived plasma cell-free DNA fraction for antibody-mediated rejection in post renal transplant recipients: A prospective observational study. Front. Immunol. 11, 342. 10.3389/fimmu.2020.00342 32184785 PMC7058974

